# Rapid formation of gold core–satellite nanostructures using Turkevich-synthesized satellites and dithiol linkers: the do's and don'ts for successful assembly†

**DOI:** 10.1039/d4na00390j

**Published:** 2024-05-31

**Authors:** Runze Tang, Robert A. Hughes, Walker J. Tuff, Ana Corcoran, Svetlana Neretina

**Affiliations:** a College of Engineering, University of Notre Dame Notre Dame Indiana 46556 USA sneretina@nd.edu; b Department of Chemistry & Biochemistry, University of Notre Dame Notre Dame Indiana 46556 USA

## Abstract

Turkevich syntheses represent a foundational approach for forming colloids of monodisperse gold nanoparticles where the use of these structures as building blocks when forming multicomponent nanoassemblies is pervasive. The core–satellite motif, which is characterized by a central core structure onto which satellite structures are tethered, distinguishes itself in that it can realize numerous plasmonic nanogaps with nanometer scale widths. Established procedures for assembling these multicomponent structures are, to a large extent, empirically driven, time-consuming, difficult to reproduce, and in need of a strong mechanistic underpinning relating to the close-range electrostatic interactions needed to secure satellite structures onto core materials. Described herein is a rapid, repeatable procedure for assembling core–satellite structures using Turkevich-grown satellites and dithiol linkers. With this successful procedure acting as a baseline for benchmarking modified procedures, a rather complex parameter space is understood in terms of timeline requirements for various processing steps and an analysis of the factors that prove consequential to assembly. It is shown that seemingly innocuous procedures realize sparsely populated cores whereas cores initially obstructed with commonly used capping agents lead to few disruptions to satellite attachment. Once these factors are placed under control, then it is the ionic strength imposed by the reaction biproducts of the Turkevich synthesis that is the critical factor in assembly because they decide the spatial extent of the electrical double layer surrounding each colloidal nanoparticle. With this understanding, it is possible to control the ionic strength through the addition or subtraction of various ionic species and assert control over the assembly process. The work, hence, advances the rules for a robust core–satellite assembly process and, in a broader sense, contributes to the knowhow required for the precise, programmable, and controllable assembly of multicomponent systems.

## Abstract 2

Gold cores functionalized with dithiol linkers are densely populated with Turkevich-synthesized satellite nanoparticles in under thirty minutes at 100% yield using procedures that promote rapid assembly while avoiding potential pitfalls.

## Graphical abstract

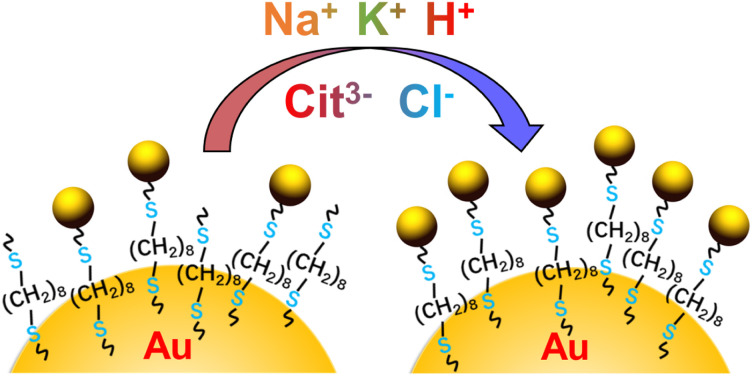

## Introduction

The rational design of functional materials through the assembly of discrete nanostructures into multicomponent systems allows for the emergence of properties that radically differ from those of the individual components.^1,2^ From this standpoint, plasmonic nanostructures represent particularly compelling building blocks because their resonant optical excitation transforms the environment immediately adjacent to the structure through the generation of intense electromagnetic near-fields,^3^ hot electrons,^4^ and heat.^5^ When such structures are placed in close proximity, plasmonic modes are fundamentally altered through hybridization,^6^ near-field intensities within the resulting nanogaps are amplified,^7^ and hot electron densities become enhanced.^8^ As such, there exists a broad-based effort to form assemblies of plasmonic structures with a scope that includes dimers,^6,9–11^ trimers,^12^ multimers,^13^ and core–satellite structures^14^ as well as large-scale assemblies with a one-,^15^ two-^16^ and three-dimensional character.^17^

The core–satellite motif, which is defined as an assembly composed of a large central core structure onto which numerous smaller satellite structures are tethered with molecular linkers, represents a fascinating architecture in that it expresses numerous plasmonic nanogaps while retaining its colloidal character. Through variations to the (i) size, shape, and composition of the core, (ii) the number, size, composition, and position of satellites, and (iii) the core–satellite and satellite–satellite spacing, there exists a design space that gives rise to an overall architectural diversity that expresses itself in the resulting structure–property relationships.^14,18–26^ Nanospheres,^14,18,19^ nanorods,^20–22^ nanocubes,^23^ nanotriangles,^24^ and nanostars^25,26^ have, for example, all been demonstrated as core materials utilizing either Au or Ag nanostructures as component materials. In another impressive demonstration, Trinh *et al.*^27^ forwarded a combinatorial approach that realizes a complex assortment of substrate-based core–satellite structures from nine different building blocks. With such variability, it is not surprising that core–satellite structures have been demonstrated in a range of applications that include biological sensing,^28–31^ bioimaging,^32,33^ targeted drug release,^30^ surface-enhanced Raman scattering (SERS),^34–36^ surface-enhanced fluorescence,^37^ temperature activated plasmonics,^22^ plasmonic rulers,^38,39^ catalysis,^40^ and colorimetric,^41–43^ chiroptical,^44^ and refractive index chemical sensors.^45,46^

Critical to the formation of core–satellite structures is the use of a molecular linker that expresses end-groups that provide a permanent bond between the core and satellite particles and whose length sets the width of the nanogap. Commonly used linkers include thiol-modified DNA,^18,36^*p*-aminothiophenol (*p*-ATP),^24,47^ and 1,8-octanedithiol (C8DT).^14,48^ The C8DT dithiol is especially attractive because it is a low-cost rigid molecule that facilitates S–Au covalent bonding, forms a self-assembled monolayer (SAM) on the Au surface, and, through variations to its chain length, allows for precise and controllable adjustments to the nanogap width.^14,49,50^ Even though the attachment of dithiol linkers to core particles is straightforward, core–satellite formation comes with the challenge of preventing the unwanted aggregation of cores. Yoon and co-workers^14^ forwarded a strategy that negated core agglomeration by affixing the core structures to a glass slide prior to dithiol functionalization. The stepwise approach, which has since been refined,^50^ begins by functionalizing glass slides with (3-aminopropyl)trimethoxysilane (APTMS) to promote nanostructure attachment when subsequently immersed into a colloid containing the core nanoparticles. The APTMS that remains exposed in the intervening areas between the core particles is then removed by immersing the glass slide into aqueous NaOH. This step is important because it prevents the attachment of standalone satellite nanoparticles to the substrate surface. As soon as this surface treatment is complete, core–satellite assembly can proceed by first functionalizing the substrate-immobilized cores in an ethanolic solution containing dithiol followed by its exposure to a colloid containing the satellite structures. Once formed, the core–satellite structures can be released from the substrate into a liquid medium through sonication. The same group has since used this technique to carry out a series of important demonstrations^27,35,51,52^ and others have both adopted and adapted this successful strategy.^36,40,43,46,48^

The satellite component of core–satellite structures is typically obtained using the Turkevich method.^53,54^ This method, which sees the reduction of tetrachloroauric acid (HAuCl_4_) with trisodium citrate (Na_3_Ct), is renowned for its ability to reliably form monodisperse spherical Au nanoparticles as an aqueous colloid using a straightforward procedure. The classical Turkevich synthesis proceeds through the rapid injection of Na_3_Ct into boiling aqueous HAuCl_4_ under constant stirring. Its seeming simplicity is, however, compromised by experimental nuances that hamper lab-to-lab reproducibility in terms of nanoparticle diameter, concentration, and size dispersity^53,55–57^ and a rather complex mechanistic underpinning^53,58–60^ where various aspects still remain contentious. Many of these issues stem from the all-important early moments of the reaction where citrate injection leads to the formation of a population of seed particles that consume a few percent of the available Au^3+^ ions. This is followed by an extended period where these seeds grow in size but where further seed formation is absent. The termination of the seed formation process, which is largely responsible for the achieved monodispersity, is generally believed to be due to the rapid rise in pH occurring in the growth solution due to the protonation of citrate upon injection. It is this rise that causes readily reduced AuCl_4_^−^ species to be transformed into less reactive [AuCl_4−*x*_(OH)_*x*_]^−^ species that are not amenable to seed nucleation events.^53,58^ Once the synthesis is complete, the remaining citrate, by acting as a nanoparticle capping agent, preserves colloidal stability. With an unwieldly parameter space that encompasses (i) precursor concentrations, molar ratios and batch volume,^58,61–63^ (ii) temperature,^54,62^ (iii) timing and order of reactant addition,^56,64,65^ (iv) pH,^53,55,58,66^ (v) mixing conditions,^56^ (vi) the duration of the synthesis^53^ and the HAuCl_4_ reflux,^55^ (vii) latent heat effects,^57^ and (viii) a host of possible additives,^53,55,56,59,64,67^ any given Turkevich reaction can yield a final product that not only differs in terms of nanoparticle size but also in the makeup of the nanoparticle capping layer as well as the biproducts remaining in the dispersion medium.

The aforementioned routes for the substrate-based assembly of core–satellite structures using Turkevich-synthesized satellites and C8DT linkers have, to a large extent, relied upon empirically derived processes where long core incubation times in both the dithiol and satellite colloid often lead to multiday processes. Herein, a methodology for the formation of core–satellite structures is demonstrated in which Au cores with pristine surfaces are populated with Turkevich-derived nanoparticles to obtain full satellite coverage in 30 min. With this highly reproducible assembly process acting as a benchmark, the influence of Turkevich-related parameters and core surface treatments on the final satellite surface coverage are elucidated. The work, hence, forwards a rapid methodology for the assembly of Au core–satellite structures and advances the fundamental understanding required to forward the design of complex multicomponent systems.

## Results

**Fig. 1:**
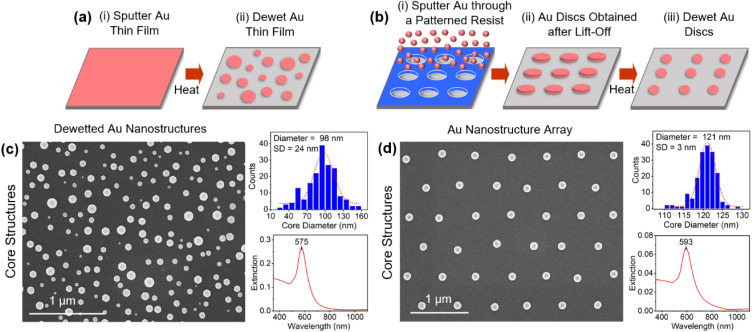
Schematics showing the procedures used to form substrate-immobilized core structures with (a) random size and position from a continuous Au film and (b) near-identically sized structures formed from lithographically-defined Au discs. Top-down SEM images, size histograms (SD = standard deviation), and extinction spectra of core structures that are (c) randomly positioned and (d) arrayed.

The overall strategy for forming core–satellite structures is an adaptation of the aforementioned approach forwarded by Yoon and co-workers^14^ in which substrate-immobilized core structures are functionalized with the C8DT dithiol linker followed by their exposure to a satellite colloid synthesized using the Turkevich method. A key difference is that the core structures are formed using a high-temperature solid-state dewetting procedure^68^ as opposed to the anchoring of spherical Au colloids to the substrate. As such, it negates the need for APTMS functionalization and its subsequent partial removal with NaOH. The dewetting procedure, which is shown schematically in Fig. 1a, sees an ultrathin Au film sputter deposited onto a low surface energy oxide substrate (*i.e.*, Al_2_O_3_) that is then exposed to a heating regimen that causes the film to reorganize into isolated equiaxed nanostructures with random size and placement.^69^

Organized surfaces of core structures with near-identical sizes were also fabricated using the procedure shown in Fig. 1b whereby nanoimprint lithography is used to define periodic arrays of polycrystalline Au discs that then undergo the same heating procedure.^70^ All of these core structures are advantageous in that they exhibit pristine Au surfaces, and as such, are unencumbered by stabilizing agents and do not require cleaning procedures that can potentially alter or damage the Au surface. At the same time, these same core structures can be subjected to various surface treatments and chemical agents to determine their impact on the assembly process (*vide infra*).

Fig. 1c and d shows top-down SEM images of the core structures obtained through both the dewetting of ultrathin films and lithographically defined features along with histograms of their size distribution and extinction spectra. Apart from weak faceting, the structures express a near-spherical geometry. Tilted-view images, however, reveal a truncation of the sphere geometry at the Au–Al_2_O_3_ interface as is common for dewetted structures (see ESI, Fig. S1†). The structures, which are well-bonded to the substrate, are single-crystals that are oriented with their [111]-axis perpendicular to the substrate.^71^ A comparison of the histograms, as anticipated, reveal a broad size distribution for the structures obtained through the dewetting of a thin film and one that is quite narrow for the array. The broad size distribution proves advantageous in this study in that it allows for a determination of the influence of the core size on core–satellite assembly where it is ensured that each core size experiences an identical chemical environment. The extinction spectra for both samples display a prominent plasmon resonance.

Over the course of this study, satellite structures were synthesized in 200 mL batches. Our standard procedure uses conditions that fall within the norms of what is typically referred to as a classical Turkevich synthesis.^53^ It should, however, be recognized that the exact conditions used can result in variations to the ionic strength of the dispersion medium as well as the nature of the capping layer that stabilizes the nanoparticles. These factors can, in turn, influence the core–satellite assembly process. As such, the synthesis is described in great detail in the Experimental section where ESI† is provided in the form of (i) a video of the all-important early stages of the synthesis (see ESI, Video S1†), (ii) a temperature profile of the growth solution (see ESI, Fig. S2†), and (iii) a timeline of key events (*e.g.*, color changes) (see ESI, Fig. S2, and S3†). Characterization of the Au colloid produced reveals a sharp plasmon resonance at 517 nm, a mean hydrodynamic diameter of 15.2 nm, and a pH of 6.0 (see ESI, Fig. S3†).

**Fig. 2:**
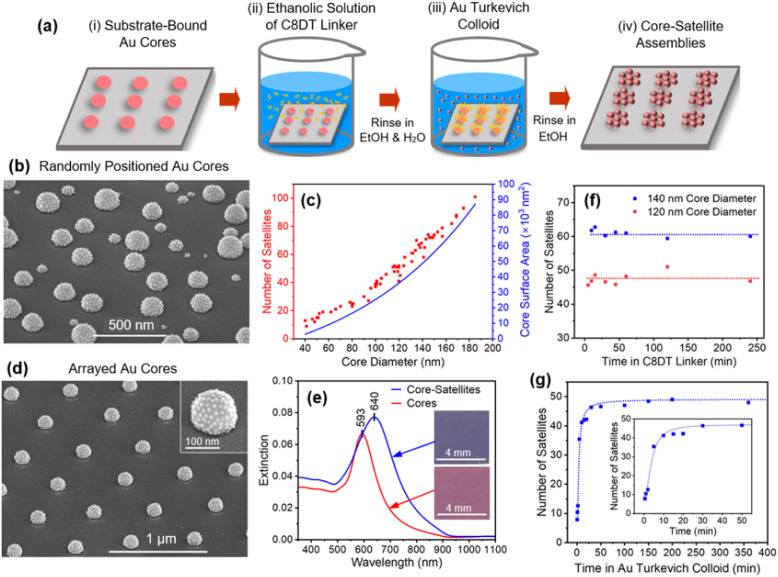
(a) Schematic of the process yielding core–satellite assemblies with maximally loaded cores in 30 min. (b) SEM image of core–satellite structures exhibiting large size variations and (c) the dependency of the core diameter on the number of satellites extracted from it (red squares) along with an estimate of core surface area that assumes a spherical shape with a 20% truncation (blue curve). (d) SEM image of an array of core–satellite structures. (e) Extinction spectra of the array showing the red shift observed when cores are populated with satellite structures. Dependencies of the number of satellites affixed to cores as the immersion time in the (f) dithiol and (g) Au colloid is varied.

Using these core and satellite structures, it is possible to form core–satellite assemblies with maximally loaded cores using the procedure shown schematically in Fig. 2a. It begins with the placement of substrate-bound cores in an ethanolic solution of the C8DT linker (1.5 mL, 0.1 mM) for 1 min. Upon removal, the sample is rinsed with ethanol (EtOH) and H_2_O to remove excess linker molecules and then inserted into the Au colloid (2 mL). Throughout this dithiol-to-colloid transfer, the functionalized surface remains wet. After 60 min, the sample is removed from the colloid whereupon it is rinsed in EtOH and dried under a N_2_ gas flow. It should be noted that the assembly process occurs in an environment where the number of Au particles within the 2 mL colloid (*ca.* 10^13^) greatly exceeds the number of cores (*ca.* 10^9^). As such, each assembly process uses less than 0.5% of the colloid and, if desired, could be reused multiple times without any overt affect upon the assembly process. The reuse of colloids did not occur in the current study.

Fig. 2b shows an SEM image of the core–satellite assemblies where it is apparent that every core structure, regardless of its size, is densely populated with satellites. The overall morphology is one that is intrinsically asymmetric since satellite attachment is forbidden on the underside of the core. Also noteworthy is that the exposed substrate remains free of satellite particles, indicating that the dithiol linker selectively binds to the Au surface. This represents an advantageous property of dithiols since other commonly used linkers, such as P4VP (poly(4-vinylpyridine)), do not express this selectivity (see ESI, Fig. S4†). SEM images were analyzed to obtain the number of satellites affixed to a core as a function of the core diameter. Fig. 2c shows this dependency together with that of the estimated core surface area where it is evident that both follow a similar trendline. It, therefore, follows that satellite particles, on average, have approximately the same spacing as the core size increases. When formed as arrays (Fig. 2d), the core–satellite structures show a high degree of uniformity even when examining areas that are more than a millimeter apart. Fig. 2e shows the extinction spectra for core structures and the same core structures after being populated with satellites. Observed is a 47 nm red shift that in optical images appears as a transition from pink to violet (Fig. 2e, inset).

Fig. 2f shows the results derived from a series of experiments where the immersion time for both 120 and 140 nm diameter cores in the C8DT linker is varied as the core–satellite assembly time is held at a constant value of 60 min. The data reveals that the functionalization of Au by C8DT is a rapid process to the extent that it proves difficult to induce a decline in particle attachment even when samples were quickly dipped in C8DT and rapidly rinsed. This finding is consistent with the work of Xue *et al.*^72^ who reported thiol–Au interaction times of only 3 s. A series of experiments were also carried out where the immersion time of cores in the Au colloid was varied as the core functionalization time with C8DT linker was held at a constant value of 1 min (Fig. 2g). It reveals that satellites are maximally loaded onto cores within the first 30 min. On the basis of this data, conservatively chosen values for the dithiol and colloid immersion times of 1 and 60 min were used in all standard syntheses. The overall speed of the core–satellite assembly process, which is unrivaled in the literature, is therefore a product of both the rapid functionalization of the cores with C8DT and a colloid that is amenable to rapid assembly. Taken together, these results confirm the viability of a rapid substrate-based core–satellite assembly process that is able to avoid long incubation times.

**Fig. 3:**
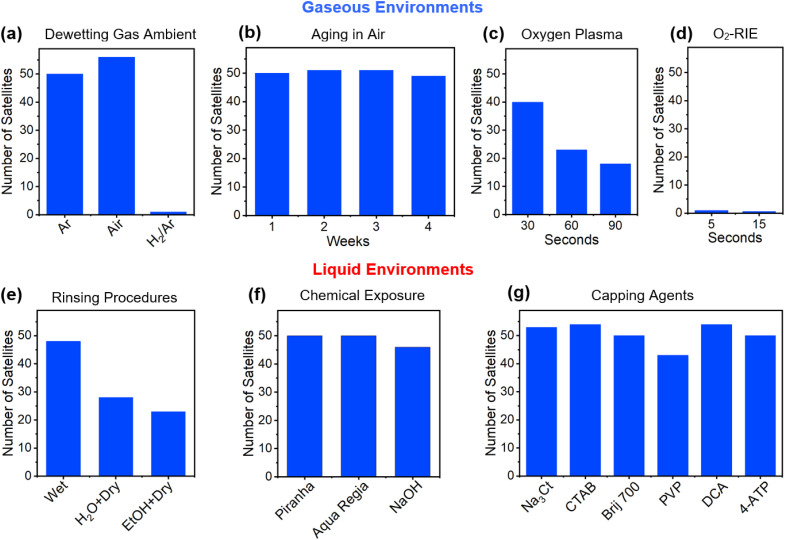
Histograms showing satellite attachment to cores that were (a) assembled in various gas ambients, (b) aged in air, (c) cleaned in an O_2_ plasma, (d) exposed to an O_2_-RIE treatment, (e) rinsed in different manners after dithiol attachment, (f) exposed to cleaning solutions, and (g) functionalized with various capping agents.

With an optimized and highly repeatable core–satellite assembly process in place, its use as a comparative benchmark for determining whether specific factors prove significant or detrimental to assembly becomes possible. With the assembly reliant on the successful functionalization of the core with the dithiol linker, any disruptions to the Au surface or the dithiol molecule before insertion into the Au colloid could prove deleterious to core–satellite assembly. As such, investigations were forwarded where satellite attachment to 120 nm diameter cores with pristine surfaces were compared to similarly sized cores produced under conditions that could potentially disrupt the assembly process. The specific conditions studied were those considered most relevant to the use of both dewetted Au structures and colloids as core materials. Investigations were, hence, dedicated to the influence of core (i) dewetting parameters, (ii) aging in air, (iii) exposures to oxygen plasmas and reactive ion etching (RIE), (iv) cleaning procedures in liquid media, (v) rinsing procedures following dithiol functionalization, and (vi) functionalization with capping agents. Fig. 3 summarizes all of the collected results in the form of histograms that detail the number of attached satellites under various conditions. These results should be viewed with the caveat that the number of satellites can vary somewhat while still being considered maximally loaded onto cores. For 120 nm diameter cores, satellite numbers for our optimal assembly conditions are consistently greater than 46.

The conditions under which the dewetting of Au films occurred were varied widely over the course of this study. Dewetting was, for the most part, carried out under an Ar gas flow but testing also occurred for air and a 10% : 90% H_2_ : Ar gas mixture. Air, while altering the core size distribution and density, displays no ill-effects on core–satellite formation. In contrast, H_2_ gas exposure led to a near-total loss in satellite attachment (Fig. 3a), a result that is likely attributable to hydrogen adsorption to the Au surface.^73^ The conditions under which dewetting occurred were also varied in terms of the film thickness deposited and heating regimen used. Even though these factors decisively alter both the core–satellite size distribution and density, the overall success of the assembly process is preserved (see ESI, Fig. S5†). Likewise, core aging experiments, which left room-temperature cores sitting out in air for up to 4 weeks, saw no decline in satellite attachment (Fig. 3b). This result indicates that our immediate use of cores in assembly processes represents an unnecessary procedure. Short exposures of the cores to excited oxygen species proved problematic in that plasma cleaning resulted in a significant decline in satellite attachment (Fig. 3c) whereas O_2_-RIE led to zero attachment even for intervals as small as 5 s (Fig. 3d). This result, which can be attributed to the adsorption of oxygen species onto the Au surface,^74,75^ is significant because such procedures are often used to clean surfaces or de-scum residues left behind by lithographic processes.

The exposure of cores to liquid-state environments prior to thiol attachment proved less problematic but caution must still be exercised. If, for example, the cores are allowed to dry following the EtOH/H_2_O rinse occurring after dithiol attachment but before entry into the Au colloid, then satellite coverage is halved (Fig. 3e). On the basis of this finding, a procedure was adopted for any process where cores are exposed to liquid media in which air exposure is avoided by requiring that the cores remain wet until the entire assembly process is complete. Using this procedure, chemical exposures of the cores to piranha solution and *aqua regia* prior to dithiol attachment were also tested because the former is commonly used to remove organic residues while brief exposures of the latter can expose a “fresh” Au surface through etching (Fig. 3f). Neither of these chemical treatments proved consequential to satellite attachment. Likewise, NaOH exposures using both the concentration and timing used by Yoon and coworkers^50^ when removing APTMS from the substrate surface resulted in near-optimum satellite coverage, a result that is consistent with their observations. The functionalization of the pristine surface of the Au cores immediately prior to dithiol functionalization through 1 h exposures to 10 mM aqueous solutions of Na_3_Ct, CTAB (cetyltrimethylammonium bromide), Brij 700, PVP (polyvinylpyrrolidone), and DCA (dicarboxyacetone) as well as an ethanolic solution of 4-ATP (4-aminothiophenol) was used to assess the influence on assembly of commonly used capping agents as well as a biproduct of the Turkevich synthesis that is known to act as a capping agent (*i.e.*, DCA).^59^ The results, shown in Fig. 3g, indicate that only PVP lies outside the range of what is considered optimal satellite attachment. The generality of these results to other capping agent concentrations and core geometries beyond those described here were not investigated. It should, however, be recognized that the concentrations used are in accordance with those commonly used in colloidal syntheses. Both PVP and DCA lead to satellite attachment to both the cores and to the substrate surface (see ESI, Fig. S6†). This finding motivated work that assessed whether any of these capping agents can independently act as linkers in an assembly process that is identical to the standard process except for the elimination of the dithiol functionalization step. It was determined that Brij 700, PVP, and 4-ATP all promote some level of satellite attachment but at levels that are substandard (see ESI, Fig. S7†). When C8DT is used in combination with these capping agents, the overall assembly process must therefore proceed (i) after a successful C8DT ligand exchange, (ii) through the commingling of C8DT with the capping agent, (iii) through bonds formed between a C8DT end group and the capping agent, or (iv) through a combination thereof.

**Fig. 4:**
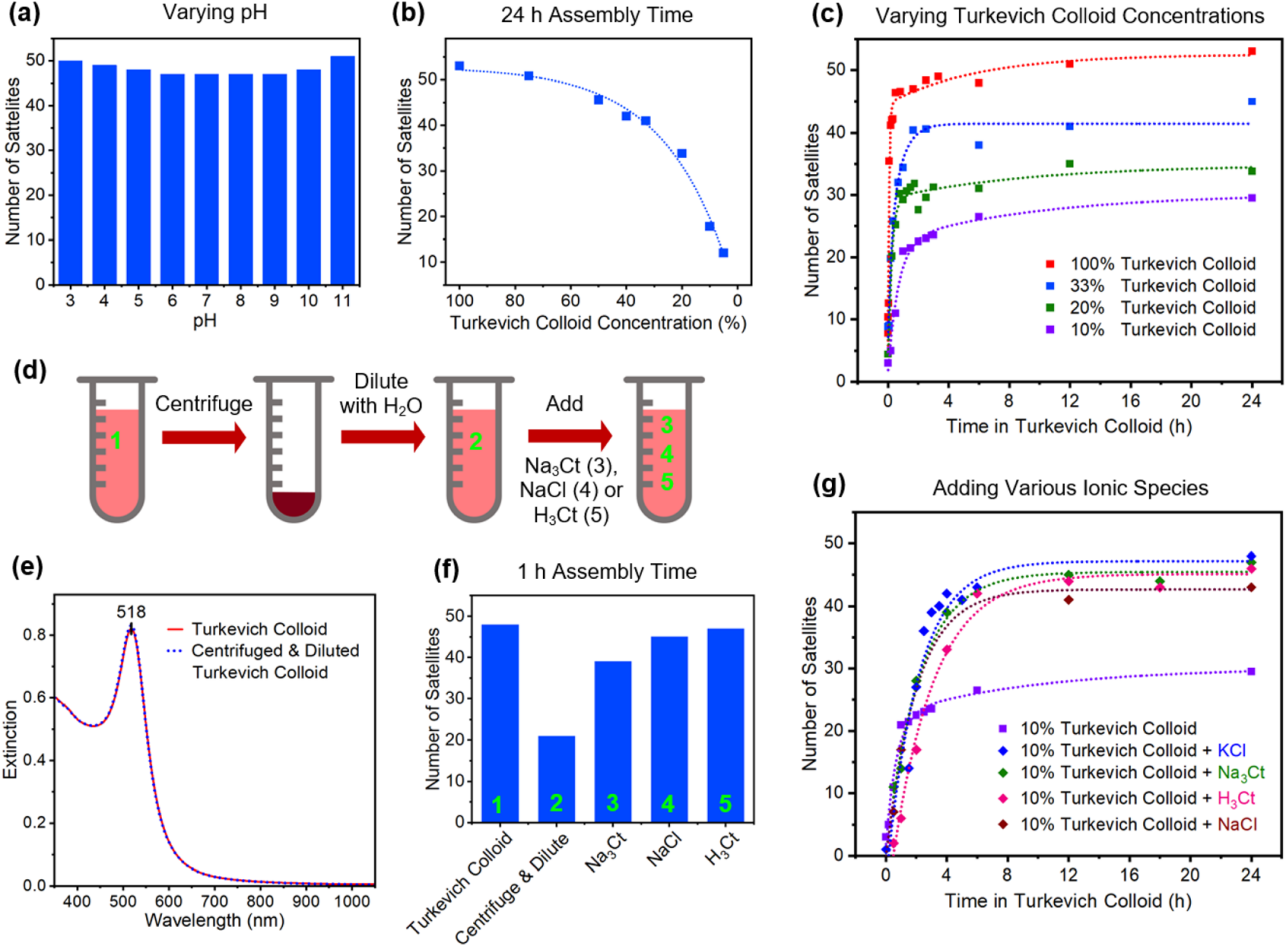
(a) Histogram showing satellite attachment to Au cores as the pH of the Turkevich colloid is varied. (b) Dependency of satellite attachment on the colloid nanoparticle concentration for assembly times of 24 h. (c) Time dependence of satellite attachment for various nanoparticle concentrations. (d) Schematic illustration of the procedure used to alter the ionic strength of the Turkevich colloid. (e) Near-identical extinction spectra for the Turkevich colloid and one where the ionic strength is reduced. (f) Histogram showing satellite attachment for the experimental scenarios shown in the schematic. (g) Time dependence of satellite attachment for a 10% nanoparticle concentration to which various ions are added.

Given that Turkevich syntheses have an unwieldly parameter space, it is difficult to assess the impact that a broad range of reaction conditions have on the assembly of core–satellite structures. Against this backdrop, investigations were carried out in which Au colloids showing optimum assembly characteristics were subjected to alterations that could potentially impact the ionic strength of the dispersion medium, alter the degree of capping, or slow the assembly process. These alterations were chosen to reflect changes that could potentially occur in the synthesis or post-synthesis processing of Turkevich colloids. Fig. 4 summarizes the results obtained when altering the satellite colloid through (i) variations to the pH, (ii) dilutions that lower nanoparticle concentration, and (iii) centrifugation followed by redispersal in various ionic media.

The pH of the Turkevich colloid was varied from 3 to 11 through additions of either HCl or NaOH that also resulted in a 10% dilution. Values outside this pH range destabilized the colloid, resulting in nanoparticle aggregation. Within this range, core–satellite assembly is resilient toward pH changes as each led to maximally loaded cores (Fig. 4a). This occurs despite the fact that the speciation of the citrate anion undergoes a progression from H_2_Ct^−^ to HCt^2−^ to Ct^3−^ over this pH range^59^ and that the ionic strength of the aqueous medium varies widely. The dilution of the colloid with H_2_O to achieve lower nanoparticle concentrations sees a steady decline in satellite attachment even when cores are exposed to the colloid for a 24 h duration (Fig. 4b). Insights into this decline were gained through an examination of the time dependence of satellite attachment for the as-synthesized colloid and for nanoparticle concentrations that are reduced to 33, 20, and 10% through dilution (Fig. 4c). For each case, the assembly is characterized by a fairly rapid rise in satellite attachment to a saturation value that persists even for long exposures to the Au nanoparticle colloid where the saturation value is dependent on the extent of the dilution. The observed decline in satellite attachment with the dilution level cannot be attributed to the availability of satellites since a 10% nanoparticle concentration still sees available satellites outnumber cores by three orders of magnitude. The decline is, therefore, attributable to a reduction in the ionic strength that accompanies each dilution.

In an effort to better understand the role of ionic strength in core–satellite assembly, experiments were carried out that resulted in the removal of both anions and cations from the Turkevich colloid followed by redispersal in various ionic liquids. The process, shown schematically in Fig. 4d, begins with the centrifugation of the Turkevich colloid followed by the removal of the supernatant containing ionic species. The precipitate is then diluted with the precise quantity of H_2_O that realizes a plasmon in the spectroscopic response that is identical to the original Turkevich colloid in terms of both peak position and extinction intensity (Fig. 4e). The product, hence, has the same concentration of nanoparticles but with a significantly reduced ionic strength due to a loss of Na^+^, H^+^, Cl^−^, and Ct ions. The ionic strength of the resulting colloid can then be controllably maneuvered through the addition of various ionic species. The results of these investigations, which are shown in Fig. 4f, indicate a 60% reduction in satellite attachment for the low ionic strength colloid for assembly times of 1 h. The result accentuates the fact that nominally identical nanoparticles in terms of both size and spectroscopic response can show stark differences in terms of their ability to realize core–satellite assemblies. The ability of the low ionic strength colloid to attain maximally loaded cores can, however, be partially restored through the addition of 0.2 mL of 40 mM Na_3_Ct to 2 mL of the ion-depleted colloid (Fig. 4f). Substituting Na_3_Ct for NaCl in quantities that realize the same Na^+^ ion addition, however, fully restores optimal assembly characteristics. This result shows that Cl^−^ additions prove more beneficial than anions derived from Na_3_Ct. The use of H_3_Ct (*i.e.*, citric acid) instead of Na_3_Ct also leads to a full restoration but its use is accompanied by the unwanted attachment of satellite particles to the substrate.

With the understanding gained, it also became possible to enhance satellite attachment for the low-concentration colloids. In a manner analogous to that used to obtain the results in Fig. 4c and g shows a time progression that compares satellite attachment for a nanoparticle concentration that is reduced to 10% through a H_2_O dilution to the same colloid when 0.1 mL of 40 mM Na_3_Ct, KCl, H_3_Ct, and NaCl is added. For all cases, satellite attachment is significantly enhanced through ion addition. As anticipated, these low concentrations require significantly longer assembly times due to fewer encounters between cores and prospective satellite components. Although the highest satellite coverage obtained is slightly below what is considered optimal, it should be noted that no optimization procedures have yet been carried out. Notwithstanding, it is clear that low concentration colloids are amenable to high levels of satellite attachment.

## Discussion

The current report forwards a procedure that, under suitable conditions, transforms what is typically a long-drawn-out core–satellite assembly process into one that is rapid, highly reproducible, and achieves a 100% yield. Through a systematic investigation of the parameter space, it is demonstrated that experimental nuances such as letting the surface dry after dithiol attachment or the well-intentioned elimination of organic contaminants through oxygen plasma cleaning represent pitfalls that can lead to sparsely populated cores. For the specific case of Turkevich-synthesized Au nanoparticles produced under the procedures described, it is shown that the reaction byproducts are a key determining factor in establishing the required ionic strength needed to promote the assembly of core–satellites structures with maximally loaded cores. Also demonstrated is the risk of post-synthesis procedures such as centrifugation followed by redispersal in that they can reduce the ionic strength of the dispersion medium to levels unsuitable for assembly. Such issues could compromise commercially available citrate-capped Au nanoparticles, making them unsuitable for core–satellite assembly process unless the as-received product is first supplemented with ions.

This study has also demonstrated the utility of using dewetted structures as a versatile platform for understanding nanoparticle assembly where the efficacy of specific procedures can be established. A key advantage is that such structures present surfaces that have not been previously compromised by ligands or surface treatments, and as such, provide surfaces that can be used to compare intrinsic assembly characteristics to that obtained for identical cores that have been functionalized or subjected to various chemical treatments. The use of this platform is not restricted to core–satellite assembly processes as it can be equally applied to the assembly of dimers, trimers, multimers, and hierarchical structures with variable size, shape, and composition. As a route to assemble substrate-based core–satellite structures, the methodology presented has both advantages and disadvantages. Drawbacks of using cores derived from a dewetting procedure as opposed to ones derived from colloidal synthesis include the (i) need to incorporate lithographic techniques if a narrow core size distribution is desired, (ii) use of a substrate that can withstand the high temperatures needed for dewetting to occur, and (iii) requirement that the linker shows no affinity for binding to the substrate if a bare substrate is to be preserved. Advantageous is that (i) the pristine surface of the Au core is amenable to a collection of synthetic techniques that are able to transform the shape of the substrate-bound structures to achieve geometries including nanoplates,^76^ nanocubes,^77,78^ nanostars,^79,80^ and Janus structures,^81^ (ii) lithographic techniques allow for the site-selective placement of core–satellite structures, (iii) core–satellite structures can be formed on surfaces at high densities (Fig. S5a†), and (iv) the assemblies are well-bonded to the substrate to the extent that they can withstand sonication. Taken together, there is the opportunity to use this platform to advance the rules for assembly, disentangle rather complex parameter spaces, and form assemblies that are unrealizable using other techniques.

The results presented herein also highlight the importance of the ionic strength in regulating assembly processes. From a mechanistic standpoint, the overall concept of core–satellite assembly is straightforward in that the functionalized core structure presents thiol end groups amenable to the formation of covalent bonds with colloidal Au nanoparticles that arrive to the surface along pathways governed by Brownian motion. This interaction is, however, complicated by the fact that citrate-capped colloidal nanoparticles and ionized dithiols both express negative charge and, as such, both the core and any incoming satellite particle are inevitably surrounded by an electrical double layer (EDL)^10^ that must be penetrated if a satellite binding event is to occur. The Debye length, which is a measure of the spatial extent of the EDL, when at large values is advantageous for maintaining colloidal stability but where lower values are beneficial when trying to promote the close encounters needed for a satellite to bind to a core structure. The ionic strength of the dispersion medium comes into play in that it is the single most important factor in determining the Debye length with larger ionic strengths realizing smaller values.^10^ In an ideal scenario, the ionic strength is high enough to allow colloidal particles to have close enough encounters with core particles to facilitate Au–dithiol bond formation but low enough to maintain colloidal stability. As more satellites are added to the core, the same EDL layers that facilitate colloidal stability make it increasingly difficult to add additional satellites. In time, encounters with the core–satellite structure become exclusively repulsive, at which point, the assembly process is self-terminating. Successful core–satellite assembly is, hence, reliant on a delicate balance between both attractive and repulsive electrostatic forces.

## Conclusions

In summary, we have forwarded an expedited procedure for forming core–satellite assemblies where the rules for obtaining cores with optimal satellite coverage are described. It has been determined that (i) exposures of the core to H_2_ or excited oxygen gas species should be avoided, (ii) core capping agents can have variable impacts ranging from being a nonfactor in the assembly to commingling with the linker molecule, and (iii) the surface of the core must remain wet after dithiol functionalization. Once suitably prepared cores are in place, then it is the ionic strength of the colloid dispersion medium that becomes the critical factor in determining whether cores are sparsely or densely populated with satellites. By reconfiguring the dispersion medium, colloids deemed unsuitable for core–satellite assembly can be revitalized. Taken together, these results highlight the utility of using substrate-immobilized nanostructures with pristine surfaces as a platform for disentangling a complex parameter space. The work, hence, advances best-practice procedures and the knowhow required for forming core–satellite nanostructures and provides a general-purpose platform for establishing protocols geared toward the formation of predeterminate nanoassemblies.

## Experimental Section

### Chemicals and materials

The Au target used for sputter depositions was fabricated from a 1 mm thick foil (Thermo Scientific) with a purity of 99.9985% using a 19 mm diameter punch and die setup. [0001]-oriented Al_2_O_3_ substrates (MTI Corp.) were diced from larger wafers to sizes of 10 mm × 5 mm × 0.65 mm for randomly positioned dewetted structures and 10 mm × 10.5 mm × 0.65 mm for arrayed structures. The reagents used in Au nanoparticle synthesis *via* the Turkevich method are hydrogen tetrachloroaurate(iii) trihydrate (HAuCl_4_·3H_2_O, 99.99% trace metal basis, Beantown Chemical), trisodium citrate dihydrate (Na_3_C_6_H_5_O_7_·2H_2_O, 99% pure, Thermo Scientific), and deionized (DI) water (VWR). 1,8-Octanedithiol (C8DT) and poly(4-vinylpyridine) (P4VP) linkers were sourced from Fisher Scientific and Sigma-Aldrich, respectively. Chemicals used to modify core and satellite structures include hydrogen peroxide (30% H_2_O_2_, Supelco), sulfuric acid (98% H_2_SO_4_, VWR), hydrochloric acid (6 M HCl, VWR), nitric acid (2 M HNO_3_, VWR), citric acid (C_6_H_8_O_7_, Thermo Scientific), cetyltrimethylammonium bromide (CTAB, Thermo Scientific), Brij 700 (Spectrum), polyvinylpyrrolidone (PVP, MW = 10 000, TCI), dicarboxyacetone (DCA, TCI), 4-aminothiophenol (4-ATP, TCI), sodium chloride (NaCl, Thermo Scientific), and potassium chloride (KCl, Sigma-Aldrich).

### Core synthesis

Substrate-bound cores were self-assembled through the solid-state dewetting of 10 nm thick sputter-deposited Au films deposited on [0001]-oriented Al_2_O_3_ substrates (*i.e.*, *C*-plane sapphire). Dewetting proceeded by placing the deposited films into an alumina crucible that was then loaded into a quartz tube furnace equipped with the fittings needed to maintain a continuous flow of ultrahigh purity Ar gas. After a 2 h purge of the air initially within the tube, the Au film was exposed to a heating regimen that saw the temperature (i) linearly ramped to 1050 °C in 60 min, (ii) held there for 30 min, and (iii) then naturally cooled to room temperature. It should be noted that, while film dewetting occurs at temperatures well below those used in this process, much higher values are required if the irregularly shaped dewetted islands are to subsequently assemble into sphere-like structures in a timely manner.^69^ The procedure used to form periodic arrays of polycrystalline Au discs followed by their assembly into single-crystal Au cores is described in detail elsewhere.^70,81^ The process yields a hexagonal pattern of 10^8^ structures over a 8 mm × 8.3 mm with a center-to-center distance of 600 nm. Apart from the aforementioned core aging experiments, the Au core structures, once assembled, remained in the flowing Ar ambient until they were subjected to the core–satellite assembly process. Chemical exposures of the cores to piranha solution (Hazard: strong oxidizer), *aqua regia* (Hazard: highly toxic and corrosive), and 1 mM NaOH (*i.e.*, Fig. 3f) occurred for 2 min, 5 s, and 6 h, respectively. RIE exposures were carried out at 20 W. Core functionalization with various capping agents (*i.e.*, Fig. 3g) used 1 h exposures at a concentration of 10 mM.

### Satellite synthesis

Turkevich syntheses begin with the preparation of an aqueous 100 mM HAuCl_4_ stock solution that, when not in use, is stored in the dark. Aqueous Na_3_Ct (12.5 mL, 34 mM) is freshly prepared immediately prior to each synthesis in a 15 mL polystyrene centrifuge tube. Once the precursors are prepared, 220 mL of DI H_2_O is added to a 500 mL flat-bottom borosilicate boiling flask resting on a magnetic hotplate stirrer (SCILOGEX MS7-H550-Pro) where stirring is initiated at 350 RPM using a 5 cm PTFE coated stir bar. Stirring at this rate leads to the formation of a central vortex that causes the H_2_O level at the edge to rise by 4 mm. A micropipette is then used to add 1.25 mL of the HAuCl_4_ stock solution to the H_2_O, leading to a clear-to-yellow color change. The solution is then brought to a boil in 14.5 min using a hotplate temperature of 350 °C. At this point, the Na_3_Ct is rapidly added directly from the centrifuge tube where the timing and placement of the poured liquid relative to the liquid surface is best observed in a video of the process (see ESI, Video S1†). Under these conditions the final Na_3_Ct/HAuCl_4_ molar ratio is 3.4. The reaction is allowed to proceed under boiling conditions for 10 min as the solution progresses through a series of color changes that sees the initial yellow hue sequentially turn clear, bluish-grey, purple, and wine red (see ESI, Fig. S3a†). The flask is then removed from the hotplate, covered with the lid to a 5 cm diameter Pyrex Petri dish, and placed on a second stir plate where it is allowed to cool naturally as stirring at 350 RPM continues overnight. The synthesis yields a 200 mL Au colloid, indicating a 16% liquid loss. It should be noted that the same procedure, when carried out under reflux using a Dimroth condenser, does not affect the quality of the Au nanoparticles from the standpoint of forming high-quality core–satellite structures. The Au colloid is stored in 50 mL polystyrene centrifuge tubes under dark conditions and does not exhibit any obvious aging effects over time intervals as long as 1 year. Between syntheses, the flask and stir bar are cleaned in *aqua regia* followed by a thorough rinse with DI H_2_O.

### Core–satellite assembly

Under optimized conditions, substrate-bound cores are placed at the bottom of a 10 mL beaker containing a 1.5 mL ethanolic solution of 0.1 mM C8DT that is freshly prepared before each assembly process. After a 1 min exposure, the sample is removed with tweezers and rinsed with EtOH and H_2_O using wash bottles where the stream of liquid is directed at the tweezers such that it flows over the sample and into a waste container. Each rinse lasts approximately 10 s where, at no time, is any part of the sample allowed to dry. At this point, the fully functionalized cores are placed at the bottom of 10 mL beaker containing 2 mL of the Au colloid. After a 60 min immersion, the sample containing the assembled core–satellite structures is removed and similarly rinsed in EtOH followed by drying under a N_2_ gas flow. For assembly processes using cores functionalized with various capping agents (*i.e.*, Fig. 3g) the C8DT exposure time was increased from 1 to 60 min to allow time for a ligand exchange. Any ion additions to the colloid (*i.e.*, Fig. 4f and g) were followed by at least a 10 min wait time before core insertion. It should also be noted that every core–satellite assembly process realizing poor satellite attachment was repeated multiple times to ensure the integrity of the conclusions made where, in each case, a control sample realizing optimum satellite attachment was also produced.

### Data analysis

For all cases where the number of satellites per core structure is quantized (*i.e.*, Fig. 2–4), the reported values were obtained through the analysis of high-resolution top-down SEM images of individual structures obtained using cores derived from the dewetting process. Fig. S8† provides SEM images that demonstrate that such a process can achieve accurate satellite counts. The time-intensive, cost-prohibitive, and tedious nature of this process, however, leads to a less than satisfactory statistical analysis when only a few structures are analyzed for any given experimental condition. Confidence in the data is established through the reproducibility shown by as many as 30 control samples produced over a six-month timespan and the well-defined dependencies obtained when adjusting parameters.

### Instrumentation

Core assembly processes utilized a Gatan model 681 high-resolution ion beam coater and a Lindberg Blue M tube furnace. SEM imaging was carried out using a Helios G4 Ux SEM/FIB workstation (FEI). DLS data was obtained using Microtrac MRB, Nanotrac Wave II nanoparticle size analyzer. A JASCOV-730UV-visible spectrophotometer was used for spectroscopic characterization where the extinction values obtained for Au colloids are derived from measurements taken with a 1 cm cuvette baselined with an identical H_2_O-filled cuvette. Plasma etching and RIE were carried out in a Drytek Asher (model MS-5) and SAMCO RIE-1C reactive ion etcher, respectively.

## Conflicts of interest

There are no conflicts to declare.

## Supplementary Material

NA-006-D4NA00390J-s001

NA-006-D4NA00390J-s002
