# Supplementary material for: Rapid formation of gold core–satellite nanostructures using Turkevich-synthesized satellites and dithiol linkers: the do's and don'ts for successful assembly
Source: Nanoscale Adv. 2024 May 31;6(14):3632–43. doi: 10.1039/d4na00390j (PMC11232561; doi:10.1039/d4na00390j)
Supplement: NA-006-D4NA00390J-s001 [file NA-006-D4NA00390J-s001.pdf]

Electronic Supplementary Material (ESI) for Nanoscale Advances.
This journal is © The Royal Society of Chemistry 2024

Electronic Supplementary Information

Rapid Formation of Gold Core−Satellite Nanostructures Using Turkevich-Synthesized Satellites and Dithiol Linkers: The Do's and Don'ts for Successful Assembly

Runze Tang,[a] Robert A. Hughes,[a] Walker J. Tuff,[a] Ana Corcoran[a] and Svetlana Neretina[*ab]*

[a] College of Engineering, University of Notre Dame, Notre Dame, Indiana 46556, United States

[b] Department of Chemistry & Biochemistry, University of Notre Dame, Notre Dame, Indiana 46556, United States

[*] E-mail: sneretina@nd.edu

Video S1. The accompanying video shows the first 3 min of the Turkevich synthesis where the citrate injection speed and rapid color changes are observed.

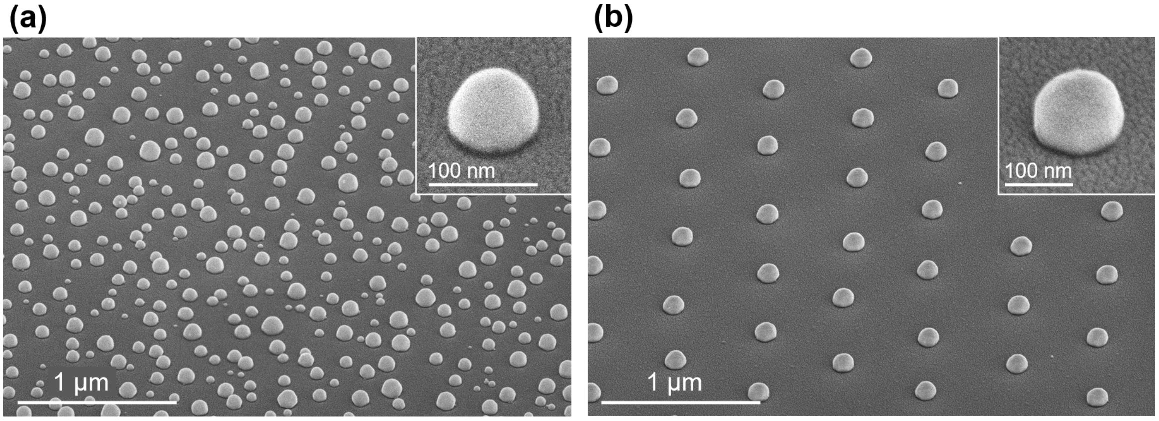

Fig. S1. Tilted-view SEM images of Au core structures arranged in (a) random and (b) arrayed configurations. It should be noted that the structures express a sphere-like geometry that is truncated at the Au−substrate interface.

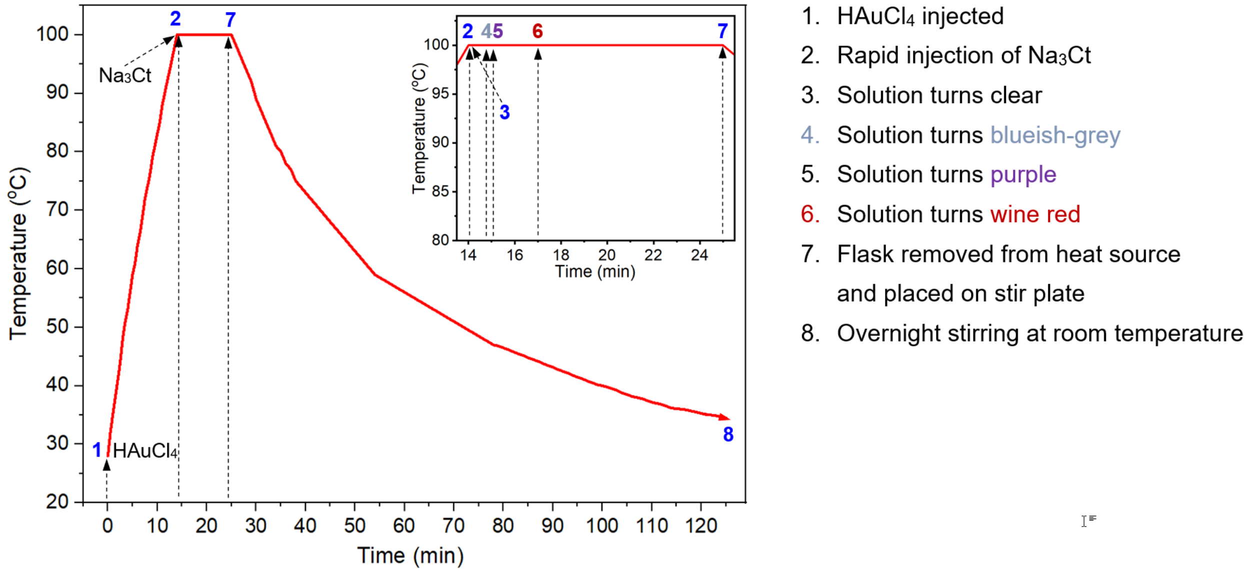

Fig. S2. Timeline of the heating regimen occurring during a Turkevich synthesis accompanied by a timeline of key events.

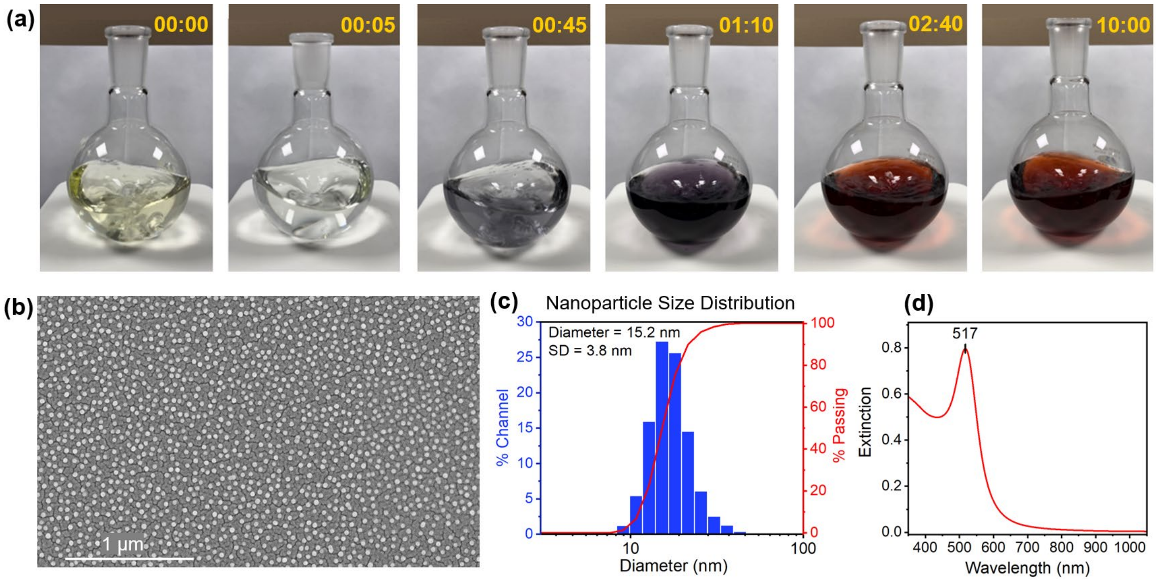

Fig. S3. (a) Images showing a time progression of the color changes occurring over the course of a Turkevich synthesis where the final product is capable of acting as satellite structures that rapidly and densely populate the core component. The labels on each figure provide time stamps for the entirety of the 10 min synthesis. Characterization of the Au colloid in the form of a (b) SEM image of the nanoparticles assembled onto a Au film, (c) histogram of the hydrodynamic diameter derived from DLS, and (d) extinction spectrum showing a prominent plasmon resonance.

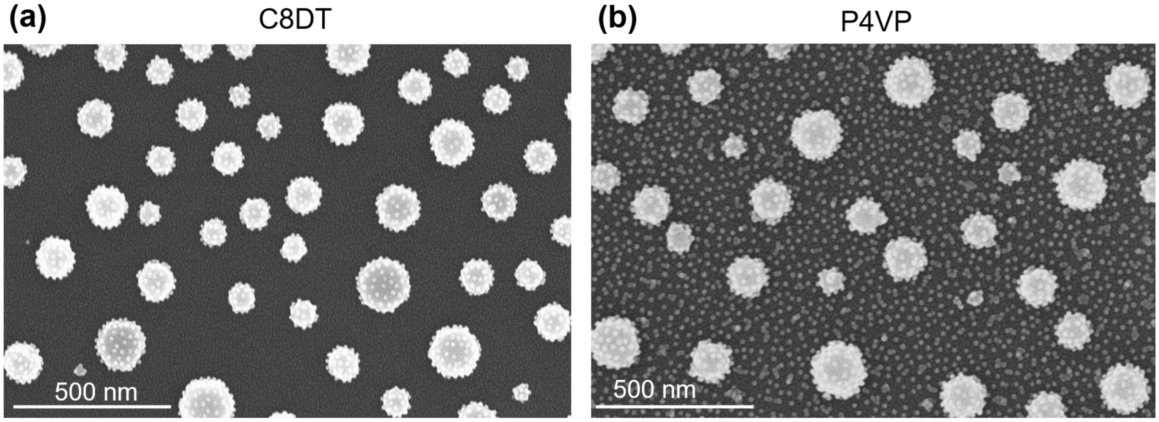

Fig. S4. SEM images of a core−satellite assemblies obtained using (a) C8DT and (b) P4VP linkers where it is apparent that the P4VP linker is disadvantageous in that core−satellite assembly is accompanied by the attachment of standalone nanoparticles to the substrate surface.

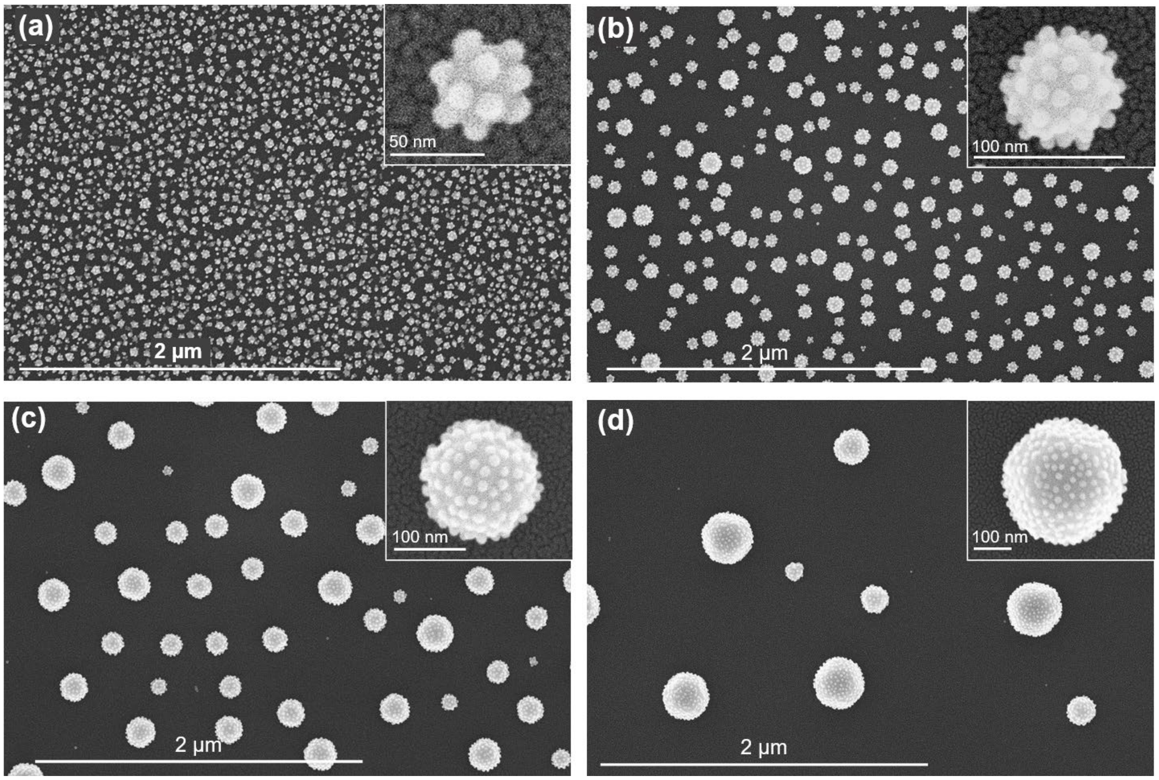

Fig. S5. SEM images of the core−satellite assemblies formed for core structures obtained when dewetting Au films with thicknesses of (a) 5.3, (b) 7.4, (c) 9.5, and (d) 11.6 nm. The data demonstrates that the Au film thickness, while changing the core size distribution and average spacing, does not impact the overall success of the assembly process.

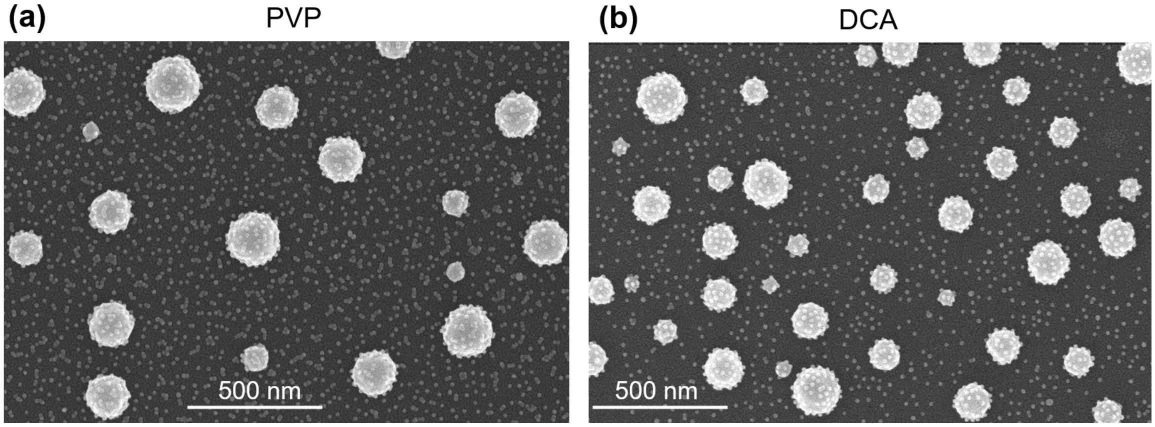

Fig. S6. SEM images of the assembled core–satellite structures when the core is functionalized with (a) PVP and (b) DCA prior to their exposure to the dithol linker. It should be noted that these capping agents promote unwanted satellite attachment to the substrate.

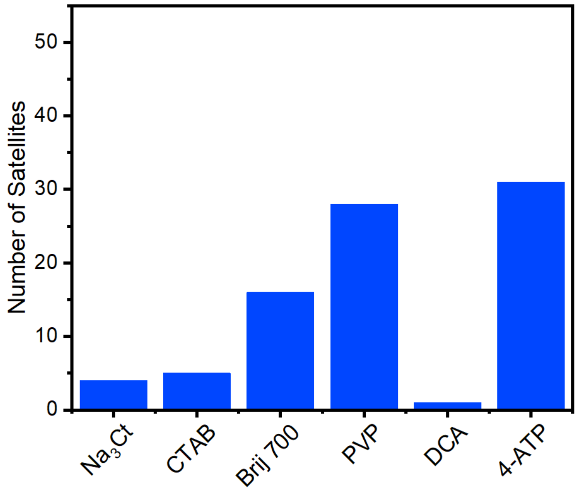

Fig. S7. Histogram showing the number of satellites that attach to the cores that have been functionalized with various capping agents but never exposed to the dithiol linker. These results indicate that Brij 700, PVP, and 4-ATP can act as linkers. It should, however, be noted that their effectiveness as linkers is poor when compared to C8DT.

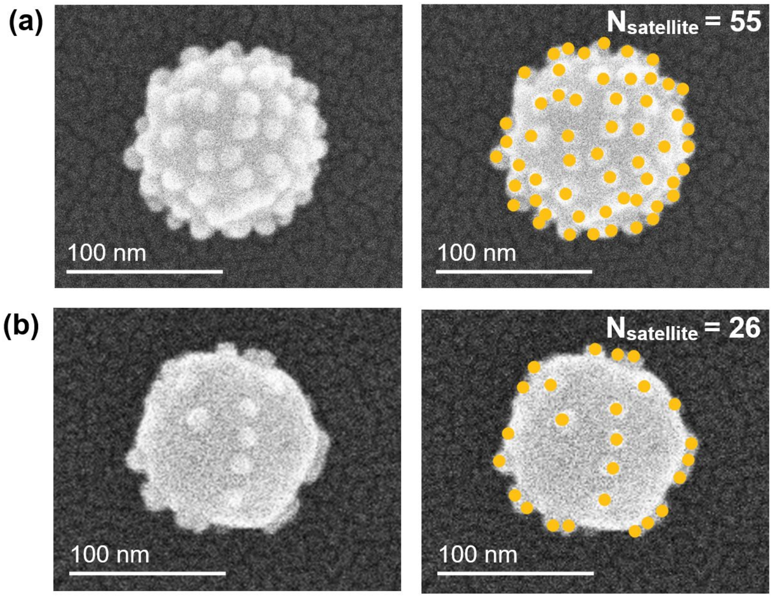

Fig. S8. High-resolution SEM images of core−satellite structures that are (a) maximally loaded and (b) sparsely populated with satellites alongside the same image where each satellite structure is denoted by a yellow dot. The figure demonstrates that such images allow for a reasonably accurate determination of the number of satellites present on individual core structures.
